# Exploration of novel biomarkers in Alzheimer’s disease based on four diagnostic models

**DOI:** 10.3389/fnagi.2023.1079433

**Published:** 2023-02-16

**Authors:** Cuihua Zou, Li Su, Mika Pan, Liechun Chen, Hepeng Li, Chun Zou, Jieqiong Xie, Xiaohua Huang, Mengru Lu, Donghua Zou

**Affiliations:** ^1^Guangxi Medical University Cancer Hospital, Nanning, Guangxi, China; ^2^Department of Neurology, The Affiliated Hospital of Youjiang Medical University for Nationalities, Baise, China; ^3^Department of Neurology, The Second Affiliated Hospital of Guangxi Medical University, Nanning, China; ^4^Clinical Research Center, The Second Affiliated Hospital of Guangxi Medical University, Nanning, China

**Keywords:** Alzheimer’s disease, random forest model, least absolute shrinkage and selection operator, logistic regression model, gradient boosting machine, biomarkers

## Abstract

**Background:**

Despite tremendous progress in diagnosis and prediction of Alzheimer’s disease (AD), the absence of treatments implies the need for further research. In this study, we screened AD biomarkers by comparing expression profiles of AD and control tissue samples and used various models to identify potential biomarkers. We further explored immune cells associated with these biomarkers that are involved in the brain microenvironment.

**Methods:**

By differential expression analysis, we identified differentially expressed genes (DEGs) of four datasets (GSE125583, GSE118553, GSE5281, GSE122063), and common expression direction of genes of four datasets were considered as intersecting DEGs, which were used to perform enrichment analysis. We then screened the intersecting pathways between the pathways identified by enrichment analysis. DEGs in intersecting pathways that had an area under the curve (AUC) > 0.7 constructed random forest, least absolute shrinkage and selection operator (LASSO), logistic regression, and gradient boosting machine models. Subsequently, using receiver operating characteristic curve (ROC) and decision curve analysis (DCA) to select an optimal diagnostic model, we obtained the feature genes. Feature genes that were regulated by differentially expressed miRNAs (AUC > 0.85) were explored further. Furthermore, using single-sample GSEA to calculate infiltration of immune cells in AD patients.

**Results:**

Screened 1855 intersecting DEGs that were involved in RAS and AMPK signaling. The LASSO model performed best among the four models. Thus, it was used as the optimal diagnostic model for ROC and DCA analyses. This obtained eight feature genes, including *ATP2B3*, *BDNF, DVL2, ITGA10, SLC6A12, SMAD4, SST,* and *TPI1*. *SLC6A12* is regulated by miR-3176. Finally, the results of ssGSEA indicated dendritic cells and plasmacytoid dendritic cells were highly infiltrated in AD patients.

**Conclusion:**

The LASSO model is the optimal diagnostic model for identifying feature genes as potential AD biomarkers, which can supply new strategies for the treatment of patients with AD.

## 1. Introduction

Alzheimer’s disease (AD) is a progressive neurodegenerative disorder, characterized by cognitive impairment and memory loss, which ultimately leads to dementia (Querfurth and LaFerla, 2010). It is mainly due to the presence of intraneuronal tau tangles or the deposition of β-amyloid (Aβ) plaques, which cause neuroinflammation, vascularization, and ultimately neuronal death (Guo et al., 2020). The risk factors for AD include age, familial inheritance, and traumatic brain injury. AD can result in vascular complications and infections (Armstrong, 2019). Up to now, a few drugs approved for the treatment of AD, which including cholinesterase inhibitors and N-methyl-d-aspartate antagonists. However, these only treat AD symptoms (Breijyeh and Karaman, 2020), and no available treatment can slow or stop the diseases, despite continuous progress in this field.

Patients with AD are usually not diagnosed in advanced stages. This implies that AD patients can be treated if it is recognized before brain injury develops. Therefore, identifying biomarkers of AD can facilitate early onset treatment or diagnosis (Auso et al., 2020). To date, 20 genetic risk loci were identified in AD, these include *APP*, *PSEN1*, and *PSEN2*, which involved the progress of early onset AD (Cuyvers and Sleegers, 2016). Homozygous loss-of-function in *TREM2*, which was previously related with autosomal recessive early onset dementia, and found to raise the risk of developing AD (Guerreiro et al., 2013). Moreover, *RBM8A* (Zou et al., 2019), noncoding miRNA-34a (miR-34a) (Jian et al., 2017), *SIRT1* (Zou et al., 2022) and *REPS1* (Luo et al., 2022) are biomarkers for early diagnosis in patients with AD. Potential pathogenic gene modules have been identified in AD (Zou et al., 2019). Furthermore, a hub gene-based signature index has been established, which may be useful for diagnosing AD (Zhou et al., 2021), but requires further exploration. Although marked progress has been made in improving the diagnosis and prediction of AD, and still a stringent need to screen new biomarkers to yield further insight into the pathogenic mechanisms underlying this disease, as well as to suggest treatment targets.

In this study, we screened AD biomarkers to lay the foundation for clinical research. We downloaded the expression profiles associated with AD and control tissue samples from the Gene Expression Omnibus (GEO) database built four models by which to identify novel biomarkers. These included random forest (RF), least absolute shrinkage and selection operator (LASSO), logistic regression, and gradient boosting machine (GBM) models. We also explored the immune cells involved associated with these biomarkers in the brain microenvironment.

## 2. Materials and methods

### 2.1. Data collection and preprocessing

Using 833 brain tissue samples in the GEO database were analyzed, including 537 AD brain tissue samples and 296 control samples. The expression profiles of the GSE125583, GSE118553, GSE5281, GSE122063, and GSE157239 were download from the GEO database (http://www.ncbi.nlm.nih.gov/geo/) (Barrett et al., 2013). GSE125583 included 219 AD and 70 controls of fusiform gyrus tissue samples, which were obtained based on the GPL16791 platform (Srinivasan et al., 2020). GSE118553 included 167 AD and 100 controls of the cerebellum, entorhinal cortex, frontal cortex, and temporal cortex tissue samples, which were obtained based on the GPL10558 platform (Patel et al., 2019). Of these, 134 asymptomatic AD patients were excluded. GSE5281 obtained 87 AD and 74 control brain tissue samples of the following brain regions: the entorhinal cortex, hippocampus, medial temporal gyrus, superior frontal gyrus, posterior cingulate cortex, primary visual cortex, and middle temporal gyrus, based on the GPL570 platform (Liang et al., 2007; Readhead et al., 2018). GSE122063 was obtained using the GPL16699 platform and included the frontal and temporal cortices from 56 AD and 44 healthy brain tissue samples, while 36 vascular dementia samples were excluded (McKay et al., 2019). The temporal cortex of eight AD patients and eight controls were obtained from GSE157239, based on the GPL21572 platform.

The expression profile of GSE125583 was normalized using the “Variance Stabilizing Transformation” function of the DESeq2 package. The expression profile of GSE118553 was normalized by “lumiExpresso” function of the Lumi package. The expression profiles of GSE5281 and GSE157239 were normalized using the “RMA” function of Affy package. Moreover, expression profile of GSE122063 was normalized using the limma package.

### 2.2. Differential expression analysis

We performed differential expression analysis screened differentially expressed genes (DEGs) of GSE118553, GSE5281, and GSE122063, and differentially expressed microRNAs (DEmiRs) of GSE157239 between AD and control brain tissue samples by limma package (Ritchie et al., 2015).^1^ Upregulated and downregulated DEGs were identified in GSE125583 using the DESeq2 package. Adjusted *p* values < 0.05 indicate notable association with AD.

### 2.3. Enrichment analyses

We obtained genes showing the same expression direction in all four datasets (GSE125583, GSE118553, GSE5281, and GSE122063) to use as intersecting genes for gene ontology (GO) and Kyoto Encyclopedia of Genes and Genomes (KEGG) analysis, using “enrichKEGG” function of the clusterProfiler package.^2^ Furthermore, gene get enrichment analysis (GSEA) was performed by expression profile of the GSE125583 dataset using the “gseKEGG” function in the clusterProfiler package. *p* < 0.05 for GO and pathway analyses were considered statistically significant.

### 2.4. Construction of four diagnostic models

We extracted the intersecting pathways of GSE125583 by GSEA and KEGG pathway analysis, and used the pROC package to calculate the area under the curve (AUC) of genes of intersecting pathways. For further selection of feature genes for inclusion in an optimal diagnostic model, AUC > 0.7 of genes in the intersecting pathways were established four diagnostic models, including the RF model, LASSO regression model, logistic regression model, and GBM model.

Random forest is a class of integrated classifiers used to construct decision-tree forests (Rigatti, 2017). Random forest has a strong predictive power and can prevent overfitting (Byeon, 2021). Therefore, we screened key genes using the RF model and obtained feature genes, by using the “randomForest” function in the randomForest package (Liaw and Wiener, 2001).

The LASSO regression model is a commonly used method of penalty regression that effectively selects feature genes from high-dimensional data and could allow effective classification in diseases (Lopez-Sanz et al., 2019; Nghiem and Potgieter, 2019). And the penalized term was selected using the 10-fold cross-validation method and the binomial bias using *λ* was calculated as important indicators to predict the ability of the diagnostic model (McEligot et al., 2020). Here, selecting the optimal genes from those with AUC > 0.7. Genes of the intersecting pathways were used to fit the LASSO regression model to screen for feature genes using the cv.glmnet function of the glmnet package (Friedman et al., 2010). Among there, the penalty function is compressed to zero, and the non-zero coefficient variable was used as the characteristic variable using the relevant formula:


$$L_{LASSO}\left(\beta \right)=-\overset{n}{\underset{i=1}{\sum }}\log\left(P\left(Yi|Xi,\beta \right)\right)+\lambda \overset{k}{\underset{k=1}{\sum }}{\left\|\beta_{k}\right\|}_{1},$$

where“||β*_k_*||_1_” refers to the L1 penalty of β_k_, is the sum of absolute values in β_k_(eliminate the intercept). The LASSO evaluates of “β” is the minimizer of the LASSO-penalized negative log likelihood function *L*_LASSO_(β). Additionally, the tuning parameter are set to the default in LASSO model.

Furthermore, logistic regression is a powerful discriminative method that clearly explains statistics and can also derive relevant classification probabilities (Zhou et al., 2021). Some studies have reported that logistic regression can assess the strongest association with outcome among various factors, which can be “adjust” for other predictor variables and factors related to outcome, without being affected by confounding factors (Tolles and Meurer, 2016). The glm function of R software was established a logistic regression model and obtained feature genes. Gene expression levels were considered as continuous predictor variables.

GBM is an iterative and correlation-based algorithm that continuously enhances the classifier through the number of user-specified iterations (Cha et al., 2021). Therefore, GBM was constructed as an AD diagnostic model, using the gbm package, to screen for feature genes of AD.

### 2.5. Receiver operating characteristic curve and decision curve analyses

To explore more powerful predictive diagnostic models, we performed our evaluations using two methods: receiver operating characteristic (ROC) curve and decision curve analyses (DCA). Using ROC analysis, the diagnostic capability of the four models were evaluated in GSE125583 and GSE118553. The more closely the AUC approximated 1, the better diagnostic efficacy was achieved. The DCA curve evaluated the diagnostic capability of models in GSE118553 and GSE125583 datasets. In this way, we obtained an optimal diagnostic model.

### 2.6. Regulation of miRNAs and feature genes in AD

We selected the feature genes that regulated the intersecting DEmiRs of GSE157239 for the TargetScan database^3^ and the miRwalk database.^4^ Subsequently, the binding sites between regulated intersecting DEmiRs with AUC values > 0.85, and feature genes were identified.

### 2.7. Expression of feature genes

We demonstrated expression level of feature genes between the AD and control groups using a heatmap and violin plot. The estimate the diagnostic capacity of feature genes in the GSE125583 dataset by AUC analysis, feature genes with AUC values exceeding 0.6 were considered to have a good predictive capacity. Finally, we determined highly expressed feature genes with the largest AUC values as hub genes.

### 2.8. Immune cell infiltration of AD patients

To compare proportion of immune cell types between AD and control samples in GSE125583, GSE118553, GSE5281, and GSE122063 datasets using single-sample GSEA (ssGSEA). By radar chart to reveal relativity between the feature genes and these immune cell types. Furthermore, positive and negative correlations of hub genes with immune cells were investigated. Besides, CIBERSORT method evaluated the abundance of infiltration of 21 immune cell types in AD patients in the GSE125583 dataset.

### 2.9. Association of hub genes with immunotherapy response

Hub genes and immunotherapy of immune genes were used to explore their relativity, immune genes of immunotherapy were grouped into the high/low expressed hub gene.

### 2.10. Statistics analysis

The Bioinforcloud platform^5^ was used to analyze all methods in this study. The adjusted *p*-values (< 0.05) for DEGs and DEmiRs were deemed to be statistically significant.

## 3. Results

### 3.1. Functional enrichment of genes in AD

**Figure 1:**
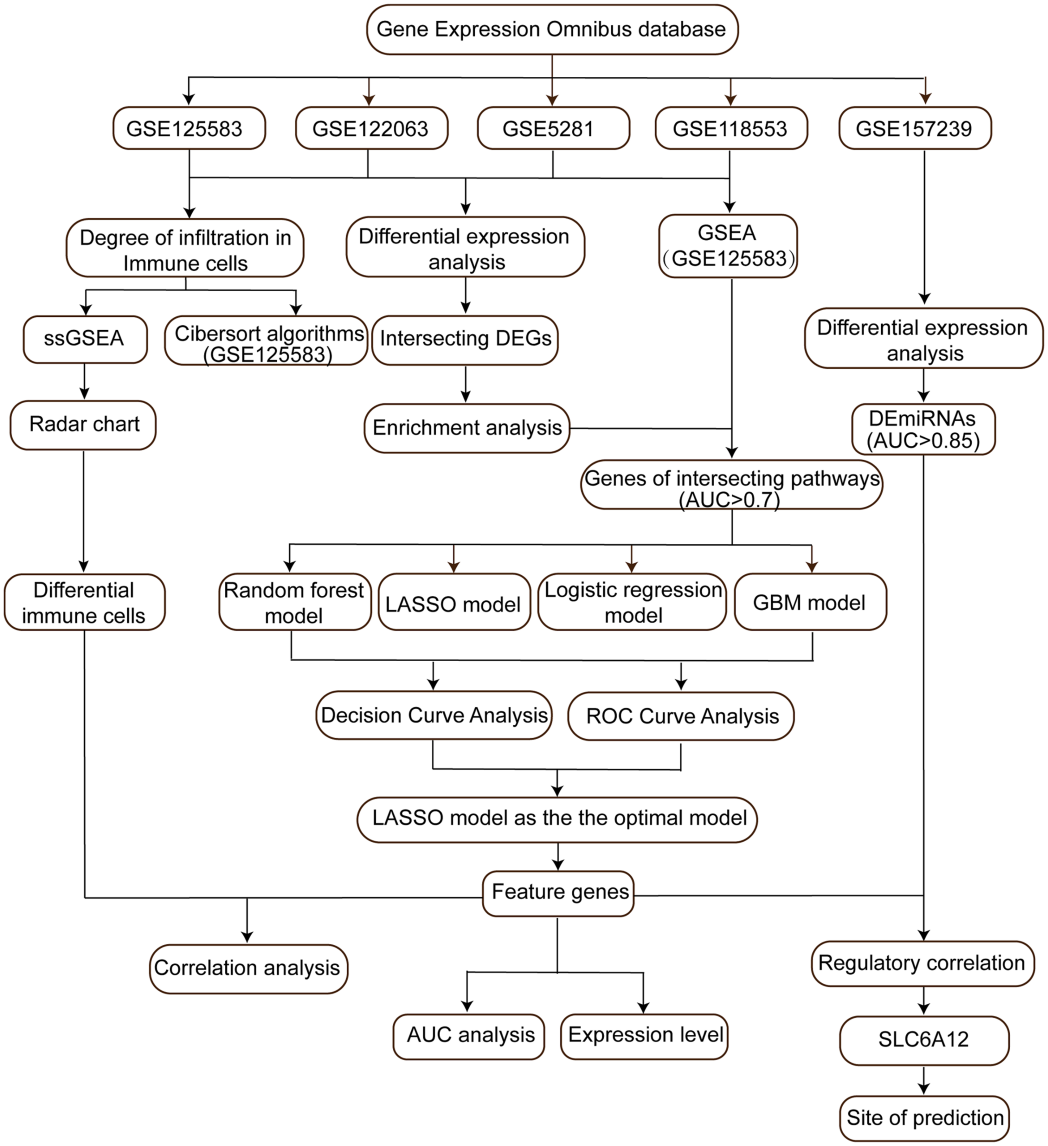
Flow chart of study. AUC, area under the curve; DEGs, differentially expressed genes; DEmiRs, differentially expressed microRNAs; GBM, gradient boosting machine; LASSO, least absolute shrinkage and selection operator; ssGSEA, single-sample gene set enrichment analysis; ROC, receiver operating characteristic curve.

**Figure 2:**
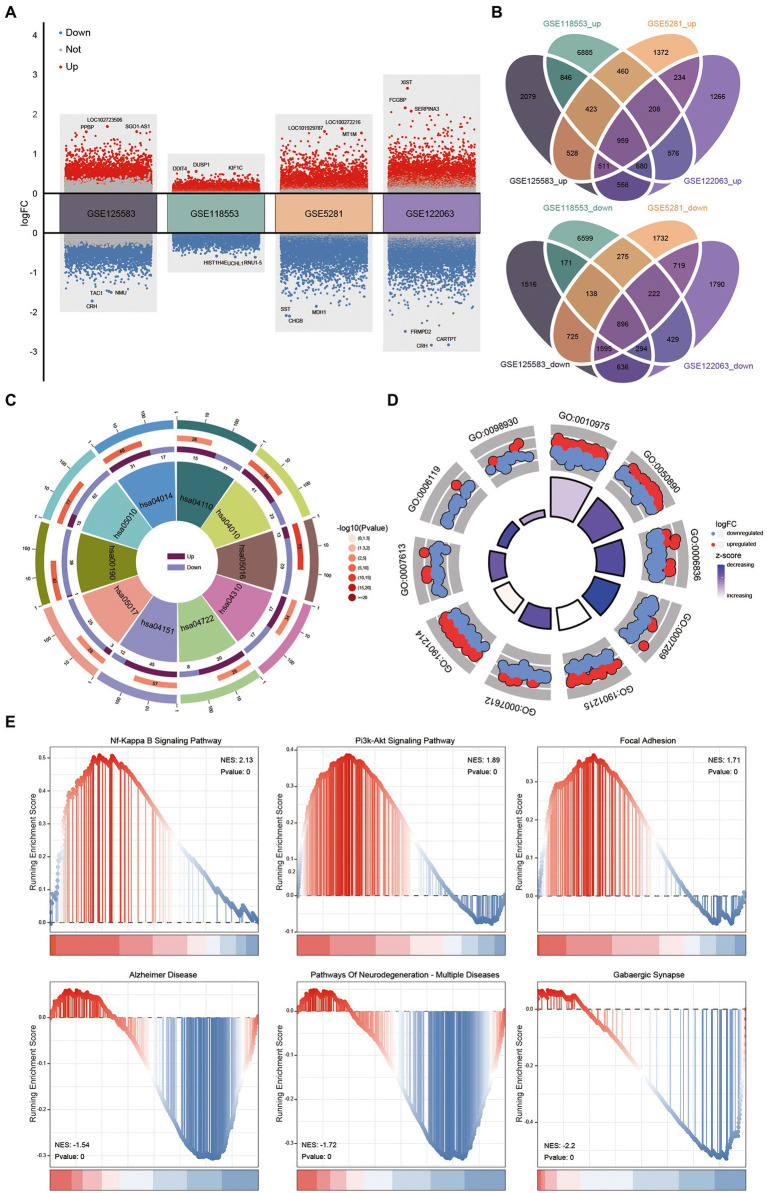
Biological function of intersected differentially expressed genes (DEGs) in Alzheimer’s disease (AD) patients and controls. **(A)** Up-/down-regulated DEGs of four datasets (GSE125583, GSE118553, GSE5281, and GSE122063), red indicates upregulated DEGs, blue indicates down-regulate DEGs. **(B)** Intersected DEGs of four datasets were obtained by Venn diagram. **(C)** The Kyoto Encyclopedia of Genes and Genomes pathways of intersecting DEGs. **(D)** Gene Ontology of intersecting DEGs were obtained using enrichment analysis. **(E)** Using gene set enrichment analysis to analysis the expressed profile of GSE125583. GO, gene ontology; NES, normalized enrichment score.

A flowchart of the study is shown in Figure 1. Identified the DEGs between AD and control tissue samples in four datasets (Figure 2A). Identified 12,555 DEGs in GSE125583, including 6,584 upregulated and 5,971 downregulated DEGs. Screened 20,061 DEGs in GSE118553, obtain 11,037 upregulated and 9,024 downregulated DEGs. In GSE5281, we identified 10,997 DEGs, including 4,695 upregulated and 6,302 downregulated DEGs. In GSE122063, 11,573 DEGs were identified, comprising 4,992 upregulated and 6,581 downregulated DEGs. As shown in Figure 2B 1,855 intersecting DEGs were found in the four datasets overall. Intersecting DEGs were enriched in RAS, neurotrophin and AMPK signaling pathways, and in the cell cycle (Figure 2C). These genes were enriched in 1,883 biological processes, including memory and learning (Figure 2D). Furthermore, the results of GSEA found that the genes identified in GSE125583 and enriched in 130 KEGG pathways. These genes were distributed in the head of pathways related to NF-kappa B signaling, P13K–Akt signaling, and focal adhesion, while they were distributed in the head in pathways related to Alzheimer’s disease, multiple neurodegeneration diseases, and GABAergic synapses (Figure 2E).

### 3.2. Feature genes of the optimal diagnostic model

**Figure 3:**
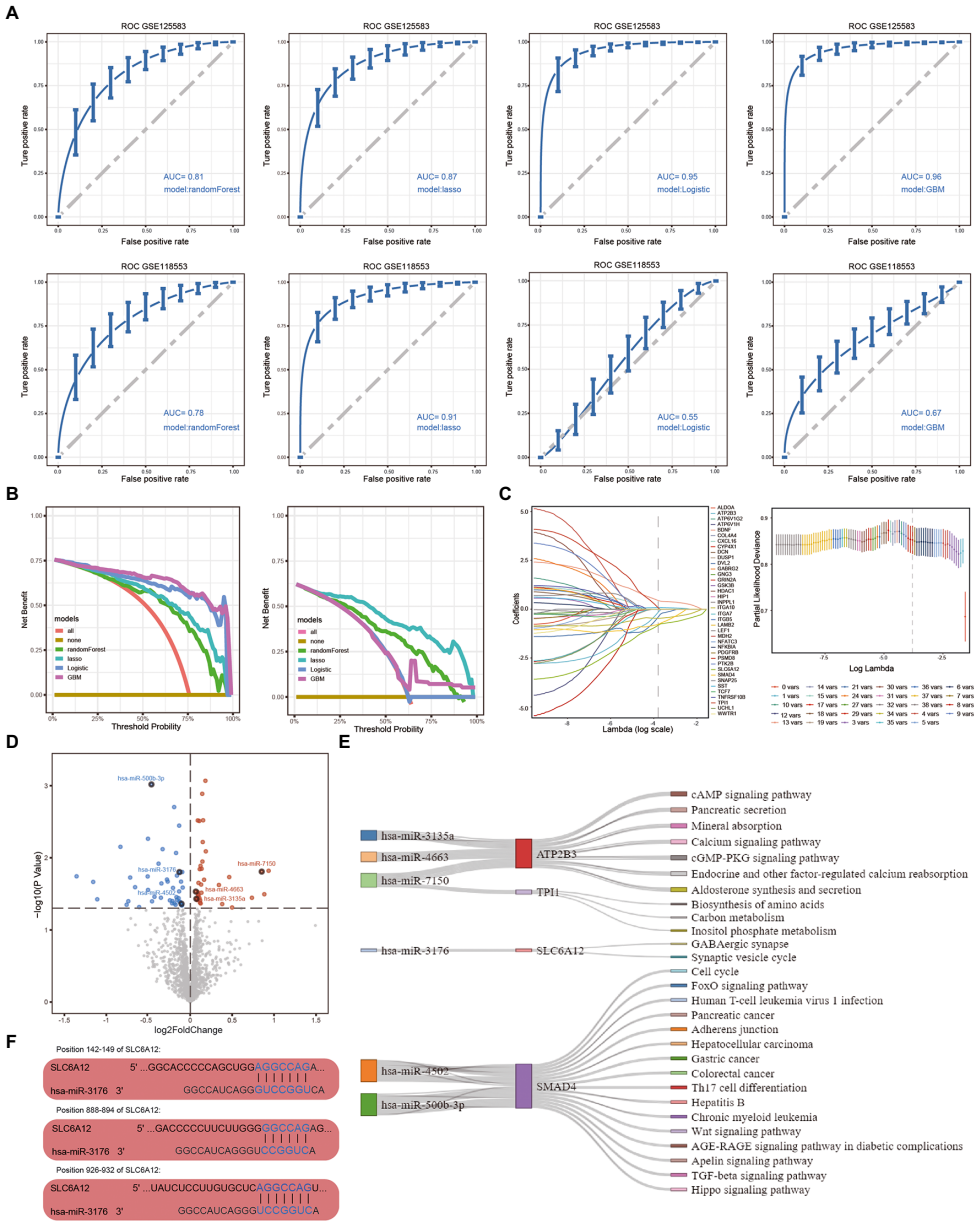
Optimal diagnostic model and feature genes obtained in Alzheimer’s disease (AD). **(A)** ROC curves showing the diagnostic performance of four models in GSE125583 and GSE118553, including random forest, LASSO, logistic regression, and GBM models. **(B)** Decision curve analysis of four models in GSE125583 and GSE118553. **(C)** The feature genes were identified by the LASSO model. **(D)** Up/downregulated DEmiRs in GSE157239. Red indicates upregulated DEmiRs, blue indicates downregulated DEmiRs. **(E)** Feature genes regulated by DEmiRs were involved in pathways. **(F)** Binding sites of the DEmiR to the hub gene. Blue font shows the position of the combination. GBM, gradient boosting machine; LASSO, least absolute shrinkage and selection operator; ROC, receiver operating characteristic curve; AUC, area under the curve; DEmiRs, differentially expressed microRNAs.

As shown in Figure 3A, we used ROC analysis to verify the diagnostic performance of four models. The results indicated that RF (AUC = 0.81), LASSO (AUC = 0.87), logistic regression (AUC = 0.95), and GBM (AUC = 0.96) all performed well in GSE125583. Furthermore, the diagnostic performance of RF (AUC = 0.78), LASSO (AUC = 0.91), logistic regression (AUC = 0.55), and GBM (AUC = 0.67) were also calculated in GSE118553. Compared with the other models, the LASSO model performed best in both the GSE125583 and GSE118553 datasets. When we used DCA to estimate the diagnostic capability of the models, we found that the logistic regression and the GBM model performed poorly in GSE125583, while the LASSO model showed optimal performance (Figure 3B). Therefore, the LASSO model was considered the optimal model for further screening of feature genes. Using this model, we obtained eight feature genes, *ATP2B3, BDNF, DVL2, ITGA10, SLC6A12, SMAD4, SST,* and *TPI1* (Figure 3C).

### 3.3. Hub genes regulated by DEmiRs in AD

The 35 upregulated and 45 downregulated DEmiRs were identified using differential expression analysis in GSE157239 (Figure 3D). We then explored which feature genes were regulated by DEmiRs. We found that *ATP2B3, TPI1, SLC6A12,* and *SMAD4* were regulated by six DEmiRs involved in the development of AD (Figure 3E). Among these, we obtained three binding sites for hsa-miR-3176 to *SLC6A12*, at positions 142–149, 888–894, and 926–932 (Figure 3F).

**Figure 4:**
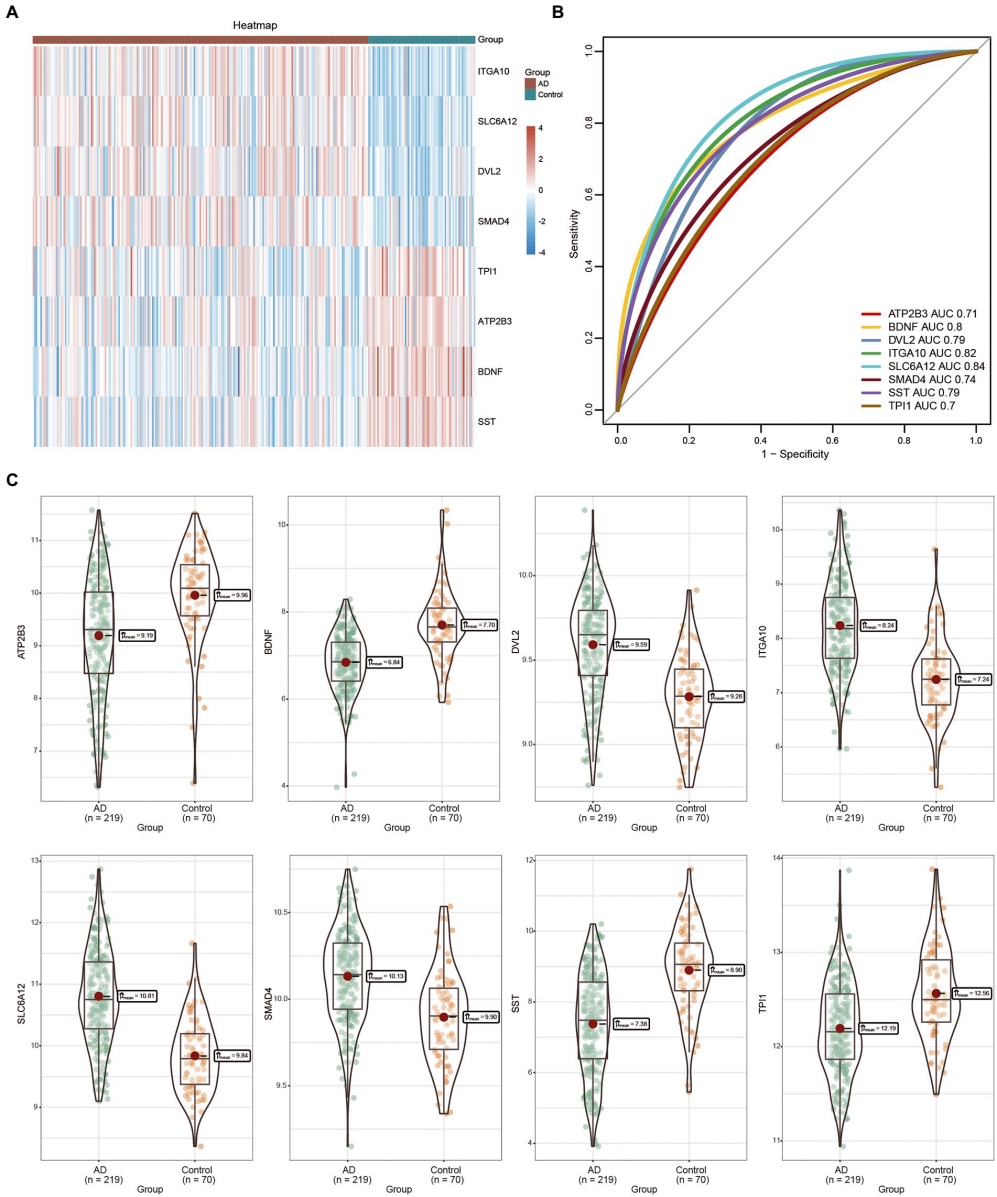
Expression of feature genes between Alzheimer’s disease (AD) and control groups. **(A)** The heatmap represents the expression of eight feature genes in AD and control groups. Red indicates high expression, blue indicates low expression. **(B)** Area under the curve of the eight feature genes. **(C)** Box diagram of the eight feature genes differentially expressed in AD and control groups. The thick black bar in the middle indicates the interquartile range, the black line extending indicates the 95% confidence interval. AD, Alzheimer’s disease; AUC, area under the curve.

Compared with the control samples, *ITGA10, SLC6A12, SMAD4,* and *DVL2* were highly expressed in the AD tissue samples (Figure 4A). The AUC values of the eight feature genes exceeded 0.7, with that of *SLC6A12* being the most significant (Figure 4B). In GSE125583, we found that *ITGA10, SLC6A12, SMAD4,* and *DVL2* were upregulated in the AD tissues, whereas *ATP2B3, BDNF, SST,* and *TPI1* were highly expressed in the control tissues (Figure 4C).

Taken together, *SLC6A12* was considered a hub gene with high expression in patients with AD, and is regulated by hsa-miR-3,176, which plays a vital role in AD.

### 3.4. Correlation of hub gene and immune cells in AD

**Figure 5:**
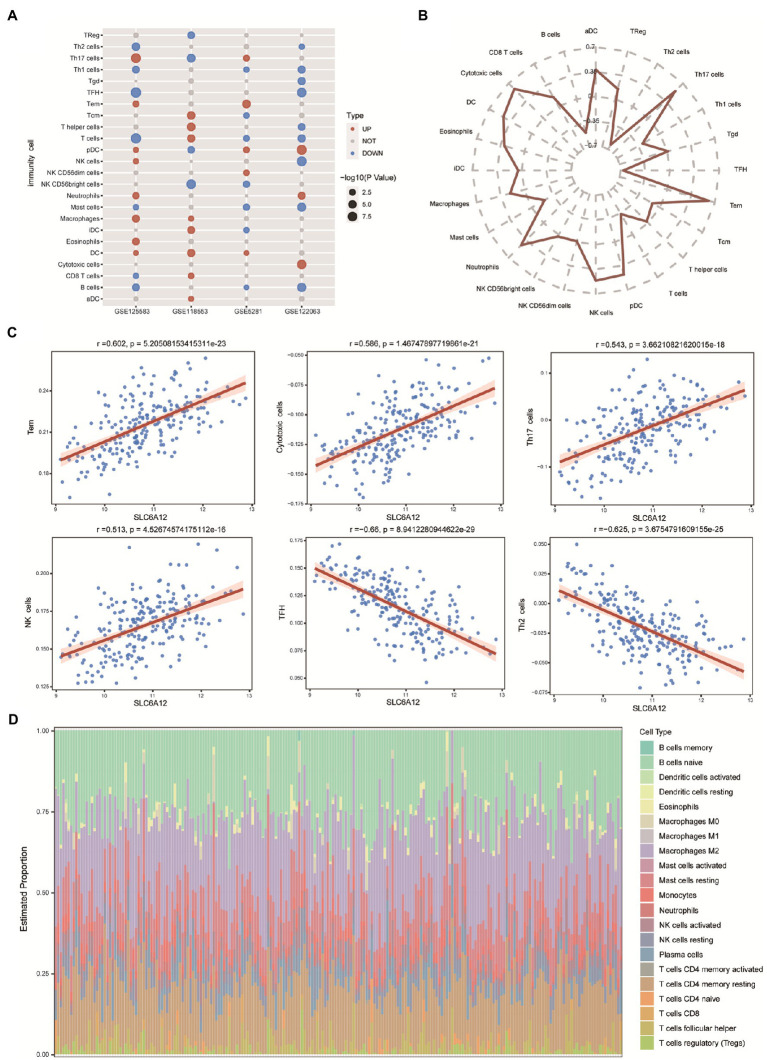
Infiltration of immune cells in Alzheimer’s disease (AD). **(A)** Infiltration of 24 immune cells in AD tissue based on expressed profiles in four datasets (GSE125583, GSE118553, GSE5281, and GSE122063). Red represents a high degree of infiltration, blue represents a low degree of infiltration. **(B)** Correlation between the feature genes and 24 immune cell types. **(C)** Correlation scatter plot displaying the hub gene and significantly positive and negative correlations. **(D)** CIBERSORT evaluation of the abundance of infiltration in GSE125583.

Using ssGSEA, we evaluated the proportion of immune cells in the brain tissues. We found that dendritic cells (DCs) and plasmacytoid dendritic cells (pDCs) were highly infiltrated in AD tissues in the three datasets (Figure 5A). We also searched the relativity between the risk scores of feature genes and 24 immune cells and found that effector memory T cells (Tem) were positively correlated with the risk scores of feature genes, yet negative correlation of follicular helper T cells (TFH) and risk score of feature genes (Figure 5B). To identify a novel immune-related gene for AD, we found that Tem, cytotoxic cells, T helper 17 (Th17) cells, natural killer (NK) cells were significantly positively correlated with *SLC6A12* expression; however, TFH and Th2 cells were significantly negatively correlated with *SLC6A12* (Figure 5C). Moreover, CIBERSORT method evaluated the abundance of immune cell infiltration. Naive B cells and macrophages M2 showed a high degree of infiltration in the brain tissues of AD patients in GSE125583 dataset (Figure 5D). These immune cells may promote the development or progression of the immune microenvironment for AD.

**Figure 6:**
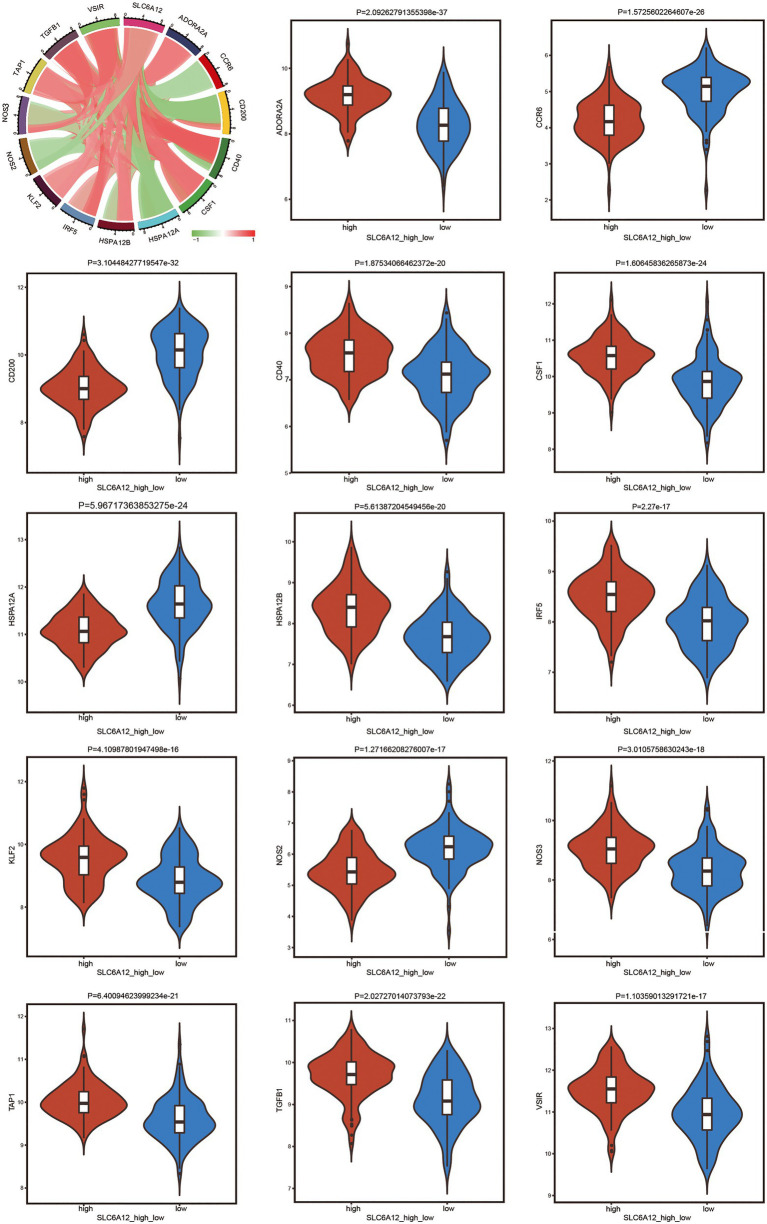
Exploring the positive and negative regulation between high or low expression of the hub gene and immune-related genes by violin plot. The thick black bar in the middle indicates the interquartile range, the black line extending indicates the 95% confidence interval.

To explore the significantly positive and negative regulation between high or low expression of *SLC6A12* and immunotherapy of immune genes, a violin plot was constructed (Figure 6). *ADORA2A, CD40, CSF1, HSPA12B, IRF5, KLF2, NOS3, TAP1, TGFB1,* and *VSIR* were positively related to the high expression of *SLC6A12*.

## 4. Discussion

Alzheimer’s disease (AD) is a neurodegenerative disease related with aging, is one of the leading causes of dementia worldwide (Zhang et al., 2021). To date, the methods used to detect AD in clinical studies have been invasive and expensive and have been unacceptable to some older individuals (Ralbovsky et al., 2019). In this study, we constructed four diagnostic models based on DEGs from AD patients, of which the LASSO model was found to be the best model for obtaining eight reliable feature genes, by both ROC and DCA. Subsequently, the binding sites between the feature genes and regulatory DEmiRs were explored, and *SLC6A12* was revealed as a key hub gene. We used ssGSEA and CIBERSORT methods to calculate the infiltrated immune cells in brain tissues of AD patients. We found a positive correlation of Tem, cytotoxic cells, Th17 cells, and NK cells with *SLC6A12* expression; however, a negative correlation of TFH and Th2 cells with *SLC6A12.* Therefore, *SLC6A12* was screened as a hub gene, showing high expression in AD patients.

A total of 1,855 intersecting DEGs were screened between AD and control brain samples in four datasets. These genes are enriched in the RAS signaling pathway, AMPK signaling pathway, and cell cycle. RAS family members were found and participated in cell growth control and metabolism, and they work with the RHO family to regulate cell cycle, expressed genes, cell transformation (Goitre et al., 2014; Song et al., 2019). The RAS/PI3K/AKT pathway may promote neuronal survival, while the PI3K/AKT/mTOR pathway shows changes in AD, Parkinson’s disease, Huntington disease (Arrazola Sastre et al., 2020). Importantly, studies found that cellular senescence acts as a major driver of age-related pathologies, such as AD, and leads to permanent cell cycle arrest (Limbad et al., 2020). However, AMPK activation can delay or prevent cellular senescence, which is important in aging (Ge et al., 2022). AMPK signaling is participated in the development of AD (Jian et al., 2021; Lin et al., 2022; Ma et al., 2022). Furthermore, cognitive functions, such as memory and learning are involved in AD (Du et al., 2018). The GSEA results indicated that the NF-κB signaling, P13K–Akt signaling, and focal adhesion were significantly upregulated in AD. NF-κB is activated in the transcription of genes participated in the inflammatory response (Vallabhapurapu and Karin, 2009). NF-κB signaling in the microglia is activated and mediates tau diffusion and tau lesion toxicity in AD (Wang et al., 2022). Furthermore, the P13K/Akt/Nrf2 pathway is considered a potential pathway for AD treatment (Meng et al., 2021). During the course of AD, focal adhesion signaling may influence neuronal viability and synaptic loss (Caltagarone et al., 2007). However, further studies on novel therapeutic targets in AD are needed. The intersecting DEGs identified in this study correlated strongly with the progress and development of AD.

Based on ROC and DCA, we found that the LASSO model was our optimal diagnostic model including eight AD-related feature genes. *ATP2B3* is a causal gene in benign aldosterone-producing adrenal lesions (Pitsava and Stratakis, 2022) and adrenocortical adenomas (Ono et al., 2020). *BDNF* encodes a neurotrophin that has been extensively studied in AD. It is also involved in the pathogenesis of brain glioblastoma (Colucci-D'Amato et al., 2020). *DVL2* may be participated in the early stages of astrocytomas (Kafka et al., 2019). The expression levels of *ITGA10*, a biomarker of type II diabetes mellitus (Wang et al., 2021), are associated with metastasis in skin cutaneous melanoma (Nurzat et al., 2021). *SLC6A12* is a key gene that promotes aggressive metastasis during ovarian cancer progression (Sung et al., 2017). *SMAD4* loss is a biomarker of squamous cell carcinoma and is used for its prognosis and prediction of treatment response (Hernandez et al., 2019). A previous study demonstrated that dysregulated *ZIP7* modulates *TPI1* expression in skeletal muscle cells and is involved in cardiovascular diseases, AD, and diabetes (Myers et al., 2013). In particular, haven studies reported that *BDNF*, *TPI1* were expressed in AD, while the other five genes were rarely reported in AD; therefore, the function of feature genes in the progress of AD needs to be validated further.

According ROC analysis, we obtained the AUC values of *SLC6A12* being the most significant comparing with the other feature genes, indicated that *SLC6A12* as a key gene and play a vital role in AD. A key regulatory relationship between low expression of hsa-miR-3176 and high expression of *SLC6A12* was found in terms of GABAergic synapses and the synaptic vesicle cycle, indicating that hsa-miR-3176 and *SLC6A12* may play a vital role in AD. To date, rarely previous study has implicated hsa-miR-3176 in cancer or in any other field (Zhang et al., 2021). GABAergic neurotransmission and modulation of GABAergic function play primary roles in AD therapy (Li et al., 2016). The synaptic vesicle cycle and GABAergic synapses involved in the mechanism of AD (Zhou et al., 2021). Thus, we considered that hsa-miR-3176 regulates high expression of *SLC6A12*, which is involved in the GABAergic synapse and synaptic vesicle cycle in the process of AD pathogenesis. Furthermore, we found that *ATP2B3*, *TPI1* and *SMAD4* were regulated with DEmiRs to play the vital role that involved in some signaling pathways for AD. Among there, low expression of *ATP2B3* regulated high expression of hsa-miR-3135a, hsa-miR-4663, and hsa-miR-7150 to promote the occurrence for cAMP and calcium signaling pathways of AD. High expressed hsa-miR-7150 was regulated the low expressed *TPI1* that involved in carbon metabolism from AD patients. High expressed *SMAD4* regulated hsa-miR-500b-3p and hsa-miR-4502 that participated in cell cycle and FOXO signaling pathway, which may be promote the development of AD. Above all, the eight feature genes may mediate the development and progression of AD, however, the specific role still needs our subsequent study.

In the present study, using ssGSEA, DCs and pDCs were found to be highly infiltrated in AD brain tissues. DCs play specific roles in the immune microenvironment during processes such as aging and in AD (Mrdjen et al., 2018). pDCs showed markedly extensive infiltration in AD (Liang et al., 2022). Furthermore, naive B cells and M2 macrophages found a high infiltrated degree in patients with AD when we used the CIBERSORT method. However, the roles of immune cells have rarely been reported in AD. Our findings suggest that immune cells infiltrate and play a key role in the immunological background of AD. Low expression of *SLC6A12,* was significantly correlated with *ADORA2A, CD40, CSF1, HSPA12B, IRF5, KLF2, NOS3, TAP1, TGFB1,* and *VSIR*. High *ADORA2A* expression may be a serum biomarker of and may take effect in AD development (Meng et al., 2020). Additionally, CD40 immunotherapy has been proposed as a new therapeutic approach for AD (Ots et al., 2022). *CSF1* (Wollmer et al., 2006), *IRF5* (Zou et al., 2012), *KLF2* (Liu et al., 2018), *NOS3* (Liu et al., 2015), and *TGFB1* (Li et al., 2018) have been associated with AD risk. Therefore, low expression of *SLC6A12* was significantly correlated with immunotherapy of immune genes that may be provided new therapeutic strategies for diagnosis with AD.

However, our study had some limitations. Although we identified eight feature genes as biomarkers of AD based on bioinformatics analysis, cell or animal experiments are still needed for verification of these findings.

## 5. Conclusion

In this study, using the optimal diagnostic LASSO model, we identified eight feature genes as biomarkers of AD: *ATP2B3, BDNF, DVL2, ITGA10, SLC6A12, SMAD4, SST,* and *TPI1*. We further identified high infiltration of DCs and pDCs as playing a vital role in the immunology of AD. These findings lay the foundation for developing new strategies for the treatment of AD patients.

## Data availability statement

The original contributions presented in the study are included in the article/supplementary material, further inquiries can be directed to the corresponding authors.

## Ethics statement

Ethical review and approval was not required for the study on human participants in accordance with the local legislation and institutional requirements. Written informed consent for participation was not required for this study in accordance with the national legislation and the institutional requirements. Written informed consent was obtained from the individual(s) for the publication of any potentially identifiable images or data included in this article.

## Author contributions

CuihuaZ, LS, MP, ML and DZ conceived and designed the study. ChunZ, LS, and MP performed analyses as well as collected and analyzed the data. All the authors prepared the figures and tables, and wrote the manuscript. All authors reviewed the manuscript and approved its submission.

## Funding

This study was supported by the Nanning Excellent Young Scientist Program and Guangxi Beibu Gulf Economic Zone Major Talent Program (RC20190103), the Basic Ability Enhancement Program for Young and Middle-age Teachers of Guangxi (2020KY13016), the Project of Baise Scientific Research and Technology Development Plan (Baike 20193107), the Scientific Research Project of Guangxi Health Commission (Z20201012, Z20210437 and Z-A20220639).

## Conflict of interest

The authors declare that the research was conducted in the absence of any commercial or financial relationships that could be construed as a potential conflict of interest.

## Publisher’s note

All claims expressed in this article are solely those of the authors and do not necessarily represent those of their affiliated organizations, or those of the publisher, the editors and the reviewers. Any product that may be evaluated in this article, or claim that may be made by its manufacturer, is not guaranteed or endorsed by the publisher.

## Footnotes

^1^ https://www.r-project.org/

^2^ https://www.r-project.org/

^3^ https://www.targetscan.org/vert_80/

^4^ http://mirwalk.umm.uni-heidelberg.de/

^5^ http://www.bioinforcloud.com

## Abbreviations

AD: Alzheimer’s disease
AUC: Area under the curve
DCA: Decision curve analysis
GBM: Gradient boosting machine
GEO: Gene Expression Omnibus
GO: Gene ontology
GSEA: Gene set enrichment analysis
KEGG: Kyoto Encyclopedia of Genes and Genomes
LASSO: Least absolute shrinkage and selection operator
NK: Natural killer
NMDA: N-methyl-d-aspartate
PD: Parkinson’s disease
RF: Random forest
ROC: Receiver operating characteristic
